# Acute camptocormia induced by olanzapine: a case report

**DOI:** 10.1186/1752-1947-4-192

**Published:** 2010-06-25

**Authors:** Florence Robert, Martial Koenig, Aurélie Robert, Stéphane Boyer, Pascal Cathébras, Jean-Philippe Camdessanché

**Affiliations:** 1 Department of Neurology, University Hospital, Saint-Etienne, France; 2 Department of Internal Medicine, University Hospital, Saint-Etienne, France; 3 Department of Radiology, University Hospital, Saint-Etienne, France; 4 Department of Psychiatry, University Hospital of Saint-Etienne, France

## Abstract

**Introduction:**

Camptocormia refers to an abnormal posture with flexion of the thoraco-lumbar spine which increases during walking and resolves in supine position. This symptom is an increasingly recognized feature of parkinsonian and dystonic disorders, but may also be caused by neuromuscular diseases. There is recent evidence that both central and peripheral mechanisms may be involved in the pathogenesis of camptocormia. We report a case of acute onset of camptocormia, a rare side effect induced by olanzapine, a second-generation atypical anti-psychotic drug with fewer extra-pyramidal side-effects, increasingly used as first line therapy for schizophrenia, delusional disorders and bipolar disorder.

**Case presentation:**

A 73-year-old Caucasian woman with no history of neuromuscular disorder, treated for chronic delusional disorder for the last ten years, received two injections of long-acting haloperidol. She was then referred for fatigue. Physical examination showed a frank parkinsonism without other abnormalities. Routine laboratory tests showed normal results, notably concerning creatine kinase level. Fatigue was attributed to haloperidol which was substituted for olanzapine. Our patient left the hospital after five days without complaint. She was admitted again three days later with acute back pain. Examination showed camptocormia and tenderness in paraspinal muscles. Creatine kinase level was elevated (2986 UI/L). Magnetic resonance imaging showed necrosis and edema in paraspinal muscles. Olanzapine was discontinued. Pain resolved quickly and muscle enzymes were normalized within ten days. Risperidone was later introduced without significant side-effect. The camptocormic posture had disappeared when the patient was seen as an out-patient one year later.

**Conclusions:**

Camptocormia is a heterogeneous syndrome of various causes. We believe that our case illustrates the need to search for paraspinal muscle damage, including drug-induced rhabdomyolysis, in patients presenting with acute-onset bent spine syndrome. Although rare, the occurrence of camptocormia induced by olanzapine must be considered.

## Introduction

Camptocormia (bent spine syndrome) refers to an abnormal posture with marked flexion of the thoraco-lumbar spine which increases during walking and resolves in supine position. Originally attributed to psychogenic disorders (war hysteria), this symptom is an increasingly recognized feature of parkinsonian and dystonic disorders, but it may also be caused by neuromuscular disorders [1,2]. A fatty degeneration of paraspinal muscles has been reported in some cases, giving support to the "myopathic theory", but there is recent evidence that both central and peripheral mechanisms may be involved in the pathogenesis of camptocormia [3,4]. We report a case of acute onset of camptocormia, with documented rhabdomyolysis and marked abnormalities on paraspinal muscular magnetic resonance imaging (MRI), probably induced by olanzapine, a second-generation anti-psychotic drug.

## Case presentation

**Figure 1:**
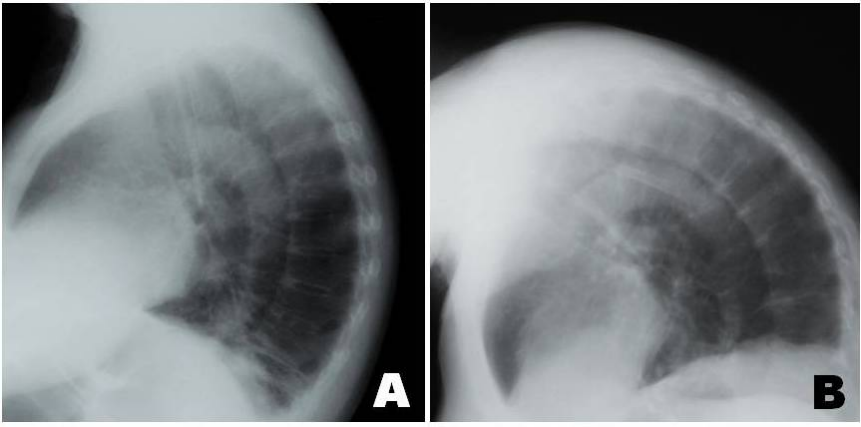
**Chest X-rays in supine position at one-week interval showing (A) porotic kyphosis and (B) camptocomic posture**.

**Figure 2:**
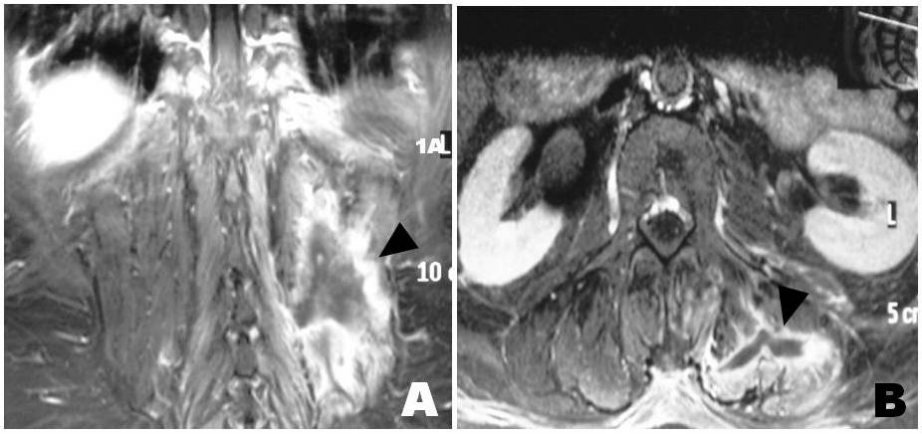
**Dorso-lumbar spine magnetic resonance imaging, (A) coronal and (B) axial post gadolinium fat saturated T1: left major para-vertebral muscle with liquid collection (necrosis) and marked contrast-enhancement (inflammation) (arrowheads)**.

A 73-year-old Caucasian woman, who had been treated for chronic delusional disorder for the last ten years, received two injections of long-acting haloperidol. She was then referred to the internal medicine department for fatigue and anorexia. Physical examination showed a frank parkinsonism without other abnormalities. Routine laboratory tests showed mild hypokalaemia, no renal dysfunction, normal muscle and liver enzymes, normal C-reactive protein value and normal thyroid tests (Table 1). The symptoms were attributed to the anti-psychotic treatment, therefore haloperidol was withdrawn, and substituted for olanzapine (5 mg/day). Our patient left the hospital after five days of olanzapine treatment without complaints. She was admitted again three days later with acute back pain. Examination showed a characteristic camptocormic posture (Figure 1) and tenderness in paraspinal muscles. Creatine-kinase level was elevated as were transaminases and C-reactive protein. Creatinine level remained normal (Table 1). MRI of the spine showed necrosis and edema in paraspinal muscles (Figure 2). Olanzapine was discontinued, pain resolved quickly and muscle enzymes were normalized within ten days (Table 1). A treatment with risperidone was later introduced without significant side-effect. The camptocormic posture had disappeared when our patient was seen as an out-patient one year later. Laboratory tests showed no abnormalities (Table 1). Our patient refused a control spinal MRI.

**Table 1:** History of the treatment and biological data (ND: not done)

	1^st ^hospitalization	2^nd ^hospitalization "admission"	2^nd ^hospitalization "10 days later"	One year later	
**Treatments**					

	**Haloperidol**	**Olanzapine**	**No treatment**	**Risperidone**	

**Biological data**					**Normal values**

Sodium	135	139	140	ND	136-146mEq/L

Potassium	2.7	3.6	4.4	ND	3.5-4.5mEq/L

Creatinine	59	61	69	ND	50-100 μmol/L

Aspartate aminotransferase	16	130	22	ND	0-45 U/L

Alanine aminotransferase	18	44	38	ND	0-45 U/L

Creatine kinase	44	2986	20	31	20-120 U/L

Creactive protein	17.4	43	3.4	ND	< 10 mg/L

Thyroid stimulating hormone	1.7	ND	ND	ND	0.5-5 mU/L

## Discussion

Olanzapine is one of the second-generation "atypical" anti-psychotic drugs, with fewer extra-pyramidal side-effects than conventional anti-psychotics, increasingly used as first line therapy for schizophrenia and delusional disorders [5]. Olanzapine has also been indicated for the treatment of bipolar disorder. Olanzapine has been held responsible for neuroleptic malignant syndrome, rhabdomyolysis or elevation of serum creatine kinase, and overdose of olanzapine is associated with acute muscle toxicity [6-8]. In our case, there is a high index of suspicion for the accountability of olanzapine in muscle damage. Based on previous reported cases, temporal connection between exposure to the drug and onset of symptoms, evidence for paraspinal muscle damage on MRI, favorable outcome after discontinuation of the drug, and lack of alternative explanation, we believe that rhabdomyolisis leading to camptocormia was probably induced by olanzapine in our patient [7]. A long term side-effect of haloperidol is less probable as this treatment was provided during ten years without problem. Neuroleptic malignant syndrome may be evoked but neither hyperthermia nor cognitive changes were observed. The combination of haloperidol and olanzapine muscle toxicity may also be discussed.

It is thus debatable whether camptocormia relates mainly to a dystonic disorder connected to Parkinson disease, or to a primary neuromuscular disorder [2]. The "muscle theory" of camptocormia has mainly been developed in Europe, and there is evidence that, at least in some cases, camptocormia relates to a primary neuromuscular disorder [1,3]. This is supported by muscle changes on computed tomography scans or spinal MRI, myopathic changes with fatty degeneration in biopsy specimens and electromyograms of the paraspinal muscles. In selected cases some improvement with steroid treatment can be observed. Camptocormia may be associated with a variety of neuromuscular disorders, such as amyotrophic lateral sclerosis, focal myopathy, inflammatory myositis including inclusion body myositis, and some other heterogeneous muscular conditions [2,3,9-12]. Laroche *et al*. (1995), basing their studies on a series of 27 patients, argued that camptocormia in older adults relates mainly to a genetically transmitted condition of muscular dystrophy or myopathy restricted to the spinal muscles [9]. However, the "central" and "peripheral" concepts of the pathogenesis of camptocormia do not necessarily contradict, as atrophy of the paraspinal muscles might be secondary to a prior action dystonia of the spine, as some recent studies have suggested [13]. Selected case reports and series indicate that both central (dysfunction in basal ganglia) and peripheral (muscle pathology) may coexist in patients with camptocormia [10,13-15].

## Conclusions

There is evidence from the literature that camptocormia is a heterogeneous syndrome of various causes. We believe that our case illustrates the need to search for paraspinal muscle damage (including drug-induced rhabdomyolysis) in patients presenting with acute-onset bent spine syndrome. Although rare, the occurrence of camptocormia induced by olanzapine must be considered.

## Consent

Written informed consent was obtained from the patient for publication of this case report and any accompanying images. A copy of the written consent is available for review by the Editor-in-Chief of this journal.

## Competing interests

The authors declare that they have no competing interests.

## Authors' contributions

FR, MK and PC interpreted the patient's data and clinical course. SB did the counseling for the psychiatric treatment. AR performed the MRI study. FR, PC and JPC were major contributors in discussing and writing the manuscript. All authors read and approved the final manuscript.
